# Regulation of Translation in Haloarchaea: 5′- and 3′-UTRs Are Essential and Have to Functionally Interact *In Vivo*

**DOI:** 10.1371/journal.pone.0004484

**Published:** 2009-02-13

**Authors:** Mariam Brenneis, Jörg Soppa

**Affiliations:** Goethe-University, Institute for Molecular Biosciences, Frankfurt, Germany; University of Edinburgh, United Kingdom

## Abstract

Recently a first genome-wide analysis of translational regulation using prokaryotic species had been performed which revealed that regulation of translational efficiency plays an important role in haloarchaea. In fact, the fractions of genes under differential growth phase-dependent translational control in the two species *Halobacterium salinarum* and *Haloferax volcanii* were as high as in eukaryotes. However, nothing is known about the mechanisms of translational regulation in archaea. Therefore, two genes exhibiting opposing directions of regulation were selected to unravel the importance of untranslated regions (UTRs) for differential translational control *in vivo*.

Differential translational regulation in exponentially growing versus stationary phase cells was studied by comparing translational efficiencies using a reporter gene system. Translational regulation was not observed when 5′-UTRs or 3′-UTRs alone were fused to the reporter gene. However, their simultaneous presence was sufficient to transfer differential translational control from the native transcript to the reporter transcript. This was true for both directions of translational control. Translational regulation was completely abolished when stem loops in the 5′-UTR were changed by mutagenesis. An “UTR-swap” experiment demonstrated that the direction of translational regulation is encoded in the 3′-UTR, not in the 5′-UTR. While much is known about 5′-UTR-dependent translational control in bacteria, the reported findings provide the first examples that both 5′- and 3′-UTRs are essential and sufficient to drive differential translational regulation in a prokaryote and therefore have to functionally interact *in vivo*. The current results indicate that 3′-UTR-dependent translational control had already evolved before capping and polyadenylation of transcripts were invented, which are essential for circularization of transcripts in eukaryotes.

## Introduction

Protein biosynthesis is comprised of three steps: initiation, elongation and termination of translation. Differential regulation of translation occurs most often at initiation because this step is rate limiting. Regulation of translation allows the cells to answer more rapidly to intracellular and extracellular changes than regulation at the transcriptional level. In humans, mistakes in translational control could be associated with diseases [1]. Translational regulation is an important mechanism involved in cell survival, differentiation, stress adoption and response to specific stimuli [2].

Until now global analyses of translational regulation have been performed only with very few species, e.g. *Saccharomyces cerevisiae*, *Arabidopsis thaliana* and human cell lines [e.g. 3]–[6]. The fraction of translationally regulated genes in these studies varied from 1% to 25% depending on species and conditions used. While more than ten studies with these three eukaryotic species have been performed, only a single genome-wide study of translational regulation in prokaryotes exists. A global analysis of translationally regulated genes of the two archaeal species *Halobacterium salinarum* and *Haloferax volcanii* revealed that 20% of all genes of the former and 6% of the latter species showed growth phase-dependent differential translational regulation [7]. This fraction is in the same range that has been found for several eukaryotic species and the study shows that translational control plays a non-negligible role for the regulation of gene expression in haloarchaea. However, nothing is known about the mechanism of translational regulation in Archaea.

Translational regulation can be achieved in various ways, e.g. key translation initiation factors can be phosphorylated or degraded [8], small noncoding RNAs can lead to gene silencing [9], riboswitches in the 5′-UTR can couple the translation of transcripts to the presence of metabolites [10], and regulatory RNA binding proteins can influence translational efficiency [10], [11].

In eukaryotes it is clear that untranslated regions (UTRs) have important biological roles and can influence key features of mRNAs, e.g. half life, intracellular localization and differential translational efficiency [12]–[16]. Examples for eukaryotic UTR elements involved in translational control are the iron response element [IRE; 11] or the cytoplasmatic polyadenylation element [CPE; 17]. RNA elments in UTRs can recruit regulatory proteins that influence translational efficiency in a stimuli-specific manner. UTR-dependent differential translational regulation is involved in metabolic regulation, stress response, development, differentiation and many other important biological processes [e.g. 13], [14], [18]–[20].

In stark contrast nearly nothing is known about the biological functions of UTRs in Archaea. The only functional role that has been characterized is the incorporation of selenocystein at stop codons in some species of methanogenic Archaea [21]. However, most Archaea do not contain selenocystein. Therefore, selenocystein incorporation is a rather specific function and more general biological roles must exist. These could include e.g. the participation in transcript half life determination or in translational control. Indeed, in a recent study it was shown that archaeal 3′-UTRs can influence transcript stabilities [22]. Furthermore, 5′-ends and the 3′-ends of 40 haloarchaeal transcripts were determined, thereby generating by far the largest experimental database of archaeal UTRs [22], including transcripts known to exhibit growth phase-dependent differential translational control [7].

The current study aimed at characterizing the role of 5′- and 3′-UTRs in translational control in *H. volcanii in vivo*. Two translationally regulated genes from the halophilic archaeon *H. volcanii* were chosen, which exhibit opposite directions of growth phase-dependent translational control. Translational regulation was monitored using a reporter gene, and the effects of various combinations of native or mutated UTRs on RNA stability and translational regulation were characterized.

## Results

### 5′-UTRs and 3′-UTRs and their role in translational regulation

**Figure 1:**
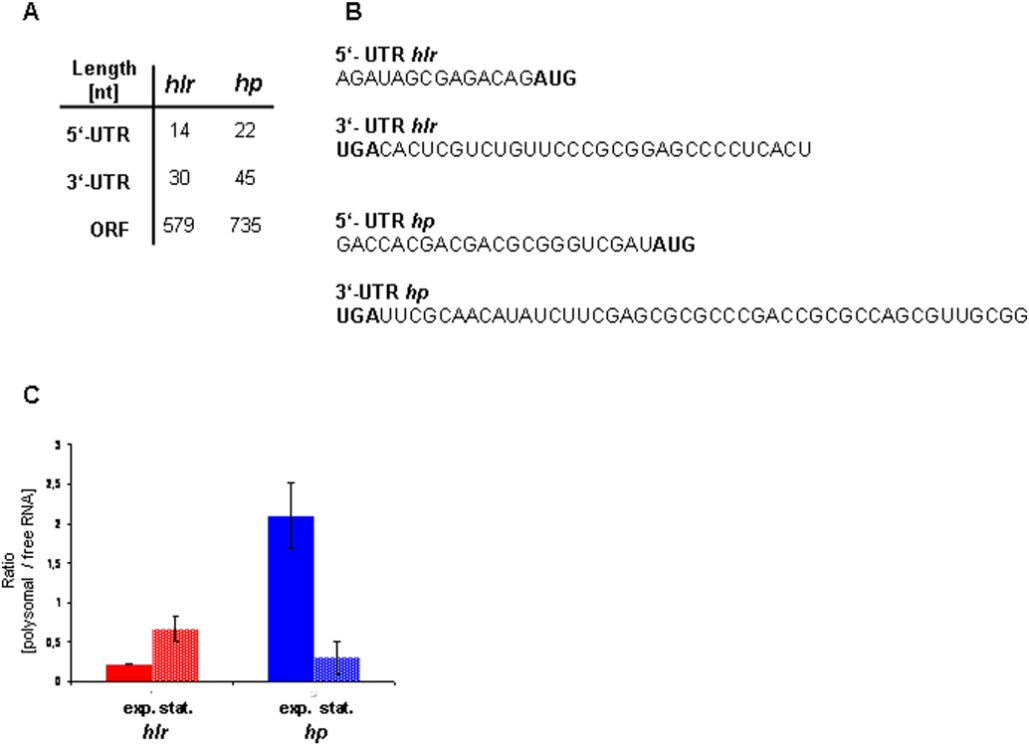
Two differentially translated *H. volcanii* genes and their UTRs. (A) The lengths of the 5′- and 3′-UTRs of the *hp* and the *hlr* transcripts are tabulated. The UTRs of the genes were determined in a prior study [22]. (B) The sequences of the 5′- and 3′-UTRs of the *hlr* and the *hp* transcripts are shown. The start as well as the stop codon of the orf are also included and printed in bold. (C) Growth phase-dependent differential translational efficiencies of the *hlr* and *hp* genes. The values were obtained by isolating free, non-translated RNAs as well as polysome-bound RNAs and their genome-wide comparison with DNA microarrays [7]. The results were normalized to the average of all genes. The ratio of polysomal to free RNA for the *hlr* and *hp* transcripts are shown for exponentially growing and stationary phase cells.

**Figure 2:**
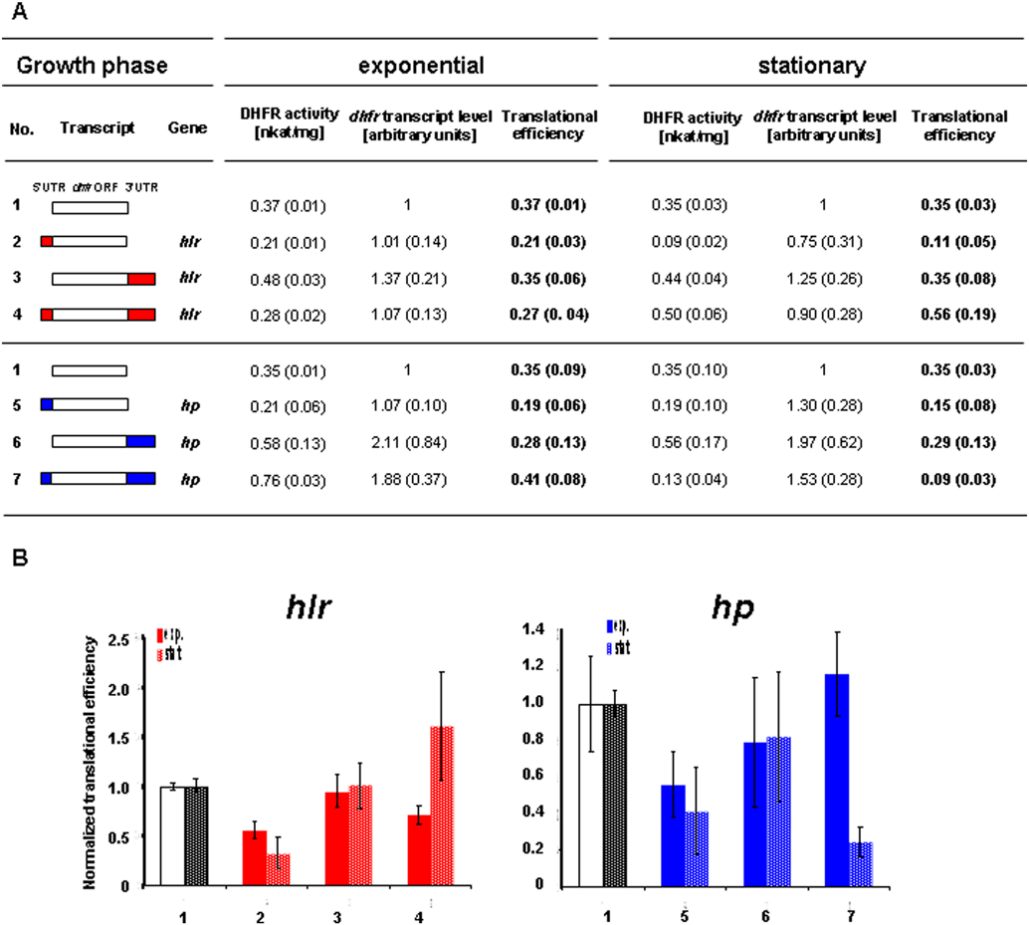
The influence of 5′UTRs and 3′UTRs on transcript stability and translational efficiency. The 5′-UTRs and the 3′-UTRs of the *hlr* gene and the *hp* gene (Figure 1A–B) were fused to the *dhfr* reporter gene alone or simultaniously. (A) The transcript fusions are shown schematically. The DHFR enzymatic activities, the *dhfr* transcript levels and the translational efficiencies of exponential and stationary growth phases are tabulated. Three biological replicates were performed and average values with standard deviations (in parenthesis) were calculated. (B) The translational efficiencies after normalization to the control transcript without UTRs (No. 1) are shown.

Two genes were chosen to characterize the *in vivo* roles of 5′- and 3′-UTRs in *H. volcanii*, i.e. the gene HVO_2837 (www.halolex.mpg.de; archaea.ucsc.edu) encoding a “hoxA like transcriptional regulator” (*hlr*) and the gene HVO_0721 encoding a “conserved hypothetical protein” (*hp*). It was shown previously that the native transcripts of both genes contain a 5′-UTR lacking a Shine-Dalgarno (SD) sequence and a 3′-UTR of average length [22]. The lengths and sequences of the UTRs are summarized in Figure 1A and B. Global analyses had revealed that the transcripts of both genes exhibit differential growth phase-dependent translational efficiencies [7]. The translational efficiency of the *hlr* transcript is down-regulated in exponential growth phase, while, in contrast, the translational efficiency of the *hp* transcript is down-regulated in stationary growth phase (Figure 1C). Previously translational regulation was determined by quantifying the fractions of free and polysome-bound transcripts using DNA microarrays, which is time-consuming and confined to native transcripts. Therefore, in the current study a reporter gene system was used to determine translational efficiencies. Transcript levels were quantified by RT-Real Time PCR and protein levels were quantified using an enzymatic test. The 5′- and 3′-UTRs of the two transcripts were fused to the *dhfr* reporter gene, either alone or simultaneously. As a control, the leaderless *dhfr* was used without its native 3′-UTR. The different transcript variants are schematically outlined in Figure 2A (all plasmids used in this study are summarized in Table 1). *H. volcanii* cultures transformed with the respective plasmids were grown to exponential growth phase (2×10^8^ cells/ml) and to stationary phase (2×10^9^ cells/ml). The *dhfr* transcript levels as well as the DHFR specific activities were determined and the translational efficiencies were calculated (Figure 2A). The results were normalized to the control transcript and are visualized in Figure 2B.

**Table 1:** Plasmids and characteristic features.

No.	Plasmid	Feature	Reference
	pNP10	negative control plasmid: shuttle vector for *E. coli* and *H. volcanii*, *neuromedinK-receptor*-gene, selection markers Nov^R^ and Amp^R^	[39]
	pSD1/M2-18	Shuttle vector for *E. coli* and *H. volcanii*, *dhfr*-gene under the control of a synthetic promoter, selection markers Nov^R^ and Amp^R^	[36]
1	pMB1	pSD1/M2-18-derivate, elimination of the native 3′-UTR of the dhfr-gene and additional nucleotides upstream of the start codons	[22]
2	pMB3	pMB1-derivate, 5′-UTR of the *hlr*-transcript	[22]
3	pMB4	pMB1-derivate, 3′-UTR of the *hlr*-transcript	[22]
4	pMB5	pMB1-derivate, 5′-UTR and 3′-UTR of the *hlr*-transcript	this study
5	pMB6	pMB1-derivate, 5′-UTR of the *hp*-transcript	[22]
6	pMB7	pMB1-derivate, 3′-UTR of the *hp*-transcript	[22]
7	pMB8	pMB1-derivate, 5′-UTR and 3′-UTR of the *hp*-transcript	this study
8	pMB24	pMB1-derivate, stabilized 5′-UTR, 3′-UTR of the *hp*-transcript	this study
9	pMB25	pMB1-derivate, destabilized 5′-UTR, 3′-UTR of the *hp*-transcript	this study
10	pMB23	pMB1-derivate, 5′-UTR of the *hp*-transcript, 3′-UTR of the *hlr*-transcript	this study
11	pMB22	pMB1-derivate, 5′-UTR of the *hlr*-transcript, 3′-UTR of the *hp*-transcript	this study

The 5′-UTRs alone had no influence on the average transcript level compared to the control variant (No. 2 and No. 5 in Figure 2A). However, fusion of the 3′-UTRs enhanced transcript abundance slightly by factors of about 1.3 and 2, respectively, in exponential as well as in stationary phase (No. 3 and No. 6 in Figure 2A). This is most likely caused by enhanced transcript stability, because promoter and 5′-part of the transcripts were identical to the control and thus identical transcription rates can be assumed.

In both cases fusion of the 5′-UTR to the reporter transcript reduced the translational efficiency by a factor of about two (No. 2 and No. 5). However, no translational regulation could be observed. Fusion of the 3′-UTRs to the reporter transcript neither influenced translational efficiency nor induced translational regulation (No. 3 and No. 6). In contrast, if 5′- and 3′-UTR were fused simultaneously to the reporter transcript growth phase dependent translational regulation was observed (No. 4 and No. 7). Translation of the *hlr* transcript was repressed by a factor of about two in the exponential growth phase, while translation of the *hp* mRNA was repressed by a factor of five in stationary phase. The direction of growth phase-dependent translational regulation in both cases was identical to that observed with the native transcripts (Figure 1C) [7], showing that the UTRs are not only essential, but also sufficient to transfer translational control to the reporter transcript.

### The structure of the 5′-UTR can influence translational efficiency and translational regulation

**Figure 3:**
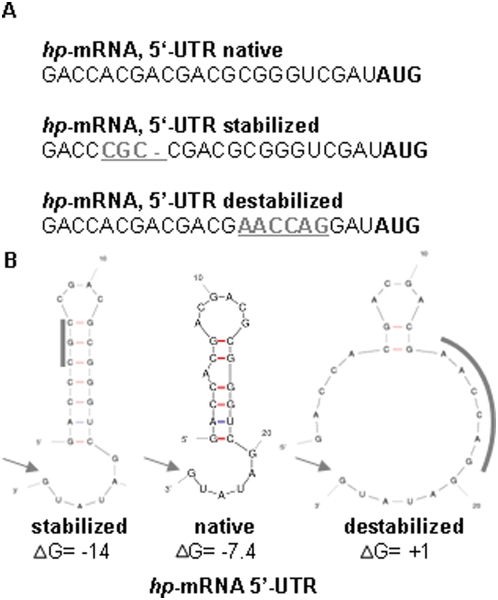
Sequences and proposed structures of native and mutated 5′-UTRs. (A) Nucleotide sequences. A putative stem loop of the native *hp* 5′-UTR was changed by *in vitro* mutagenesis. The mutated nucleotides are shown in grey and are underlined. (B) *In silico* structural analysis of the stabilized, the native, and the destabilized 5′-UTRs. The structural analysis were performed by using the mfold 3.2 program [40], [41]. Mutated nucleotides are indicated by a bar. The start codon of the orf is also included and marked by an arrow.

**Figure 4:**
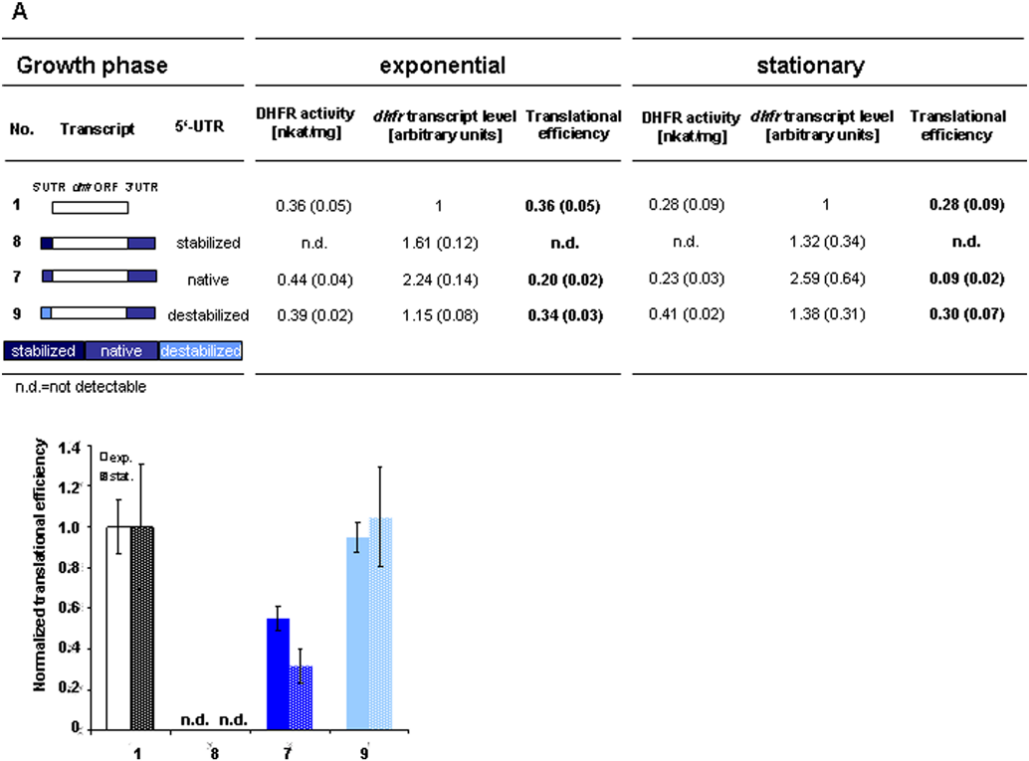
The structure of the 5′-UTR can influence translational efficiency and translational regulation. Specific putative structure elements were stabilized and destabilized in a native 5′-UTR (compare Figure 3). Translational efficiencies of the mutated versions and the wild type were determined by using the reporter gene system. (A) The transcript fusions are shown schematically. The DHFR enzymatic activities, the *dhfr* transcript levels and the translational efficiencies of exponential and stationary growth phases are tabulated. Three biological replicates were performed and average values with standard deviations (in parenthesis) were calculated. (B) The translational efficiencies after normalization to the control transcript without UTRs (No. 1) are shown.

To unravel whether secondary structures within 5′-UTRs are involved in translational regulation, both 5′-UTRs were folded *in silico*. The 5′-UTR of the *hp* transcript contained a predicted stem-loop with a ΔG of -7.4 kcal/Mol. In contrast, no convincing stem-loop was predicted for the 5′-UTR of the *hlr* transcript either alone or together with different parts of the *dhfr* open reading frame (data not shown). To verify that the predicted stem-loop might exist *in vivo* and could be involved in differential regulation, two mutated versions of the *hp* 5′-UTR with either increased or decreased stability were constructed. The sequences and predicted structures of the three versions are summarized in Figure 3. The mutated and the native 5′-UTRs were fused in combination with the native 3′-UTR to the reporter transcript. A schematic overview of the fusion variants is given in Figure 4A. Cultures containing the respective plasmids were grown to exponential phase (2×10^8^ cells/ml) and to stationary phase (2×10^9^ cells/ml). The *dhfr* transcript levels and the DHFR specific activities were determined and the translational efficiencies were calculated (Figure 4A). Normalized translational efficiencies are shown in Figure 4B.

An inverse correlation was revealed between the stabilities of the three predicted structures and the respective translational efficiencies. The stabilized stem-loop totally inhibited translation in both growth phases (No. 8), while mutational destabilization led to a constitutive high level of translation (No. 9). In both cases growth phase-dependent differential translational control was totally lost. The results support the presence of the predicted stem-loop *in vivo* and that the structure of the 5′-UTR has a strong influence on translation initiation and regulation. They indicate that the biological function of 5′-UTRs is to down-regulate the translational efficiencies in comparison to leaderless transcripts. The degree of repression of the native 5′-UTRs can be modulated in a growth phase-dependent manner, resulting in differential translational regulation.

### The 3′-UTRs determine the direction of translational regulation

**Figure 5:**
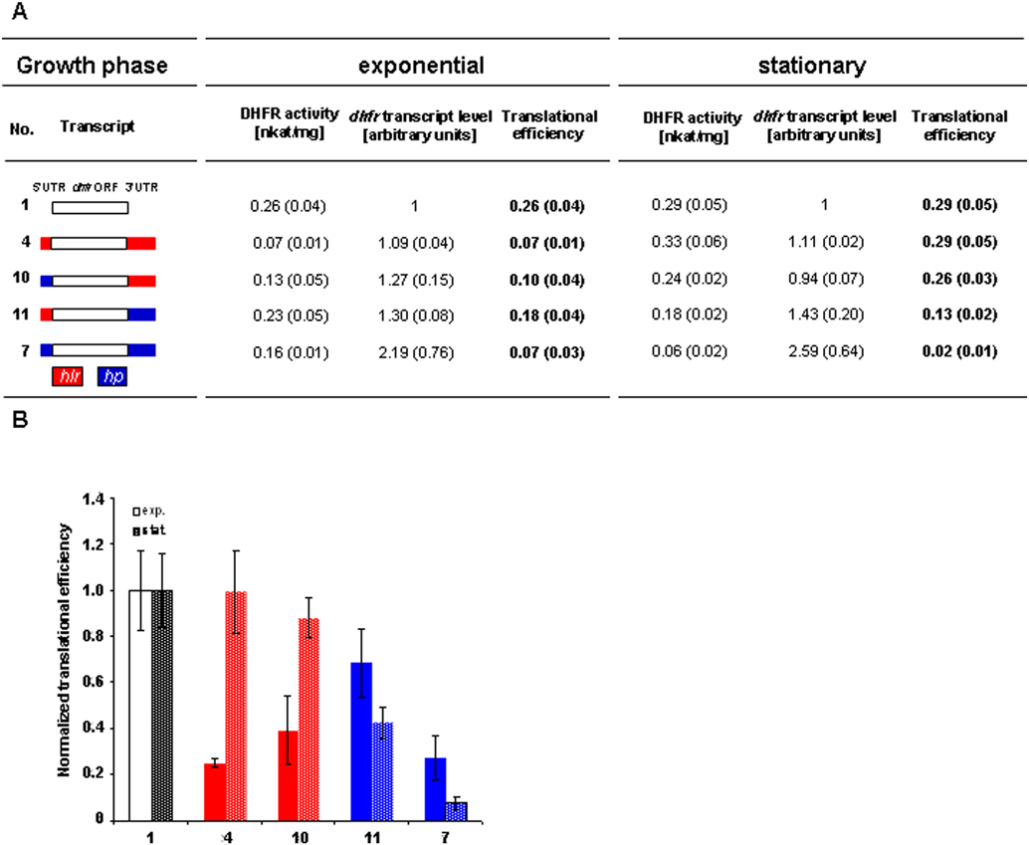
The direction of translational regulation is determined by the 3′-UTRs. Using the reporter gene system different combinations of 5′- and 3′-UTRs of the *hlr* and the *hp* transcript were studied. (A) The transcript fusions are shown schematically. The DHFR enzymatic activities, the *dhfr* transcript levels and the translational efficiencies of exponential and stationary growth phases are tabulated. Three biological replicates were performed and average values with standard deviations (in parenthesis) were calculated. (B) The translational efficiencies after normalization to the control transcript without UTRs (No. 1) are shown.

The two genes under investigation, *hlr* and *hp*, exhibit opposite directions of growth phase-dependent translational regulation. A UTR-swap experiment was performed to identify which UTR determines regulatory direction. The constructs are shown schematically in Figure 5A. Again, *dhfr* transcript levels and DHFR specific activities were determined using cultures in exponential phase (2×10^8^ cells/ml) and in stationary phase (2×10^9^ cells/ml), and translational efficiencies were calculated (Figure 5A). Normalized translational efficiencies are visualized in Figure 5B.

In both cases it turned out that the direction of regulation was retained when the 5′-UTR was exchanged (compare No. 4 with 10 and 7 with 11). In contrast, the direction of regulation was reversed after exchange of the 3′-UTRs (compare No. 4 with 11 and 7 with 10). These results clearly show that the 3′-UTRs determine the direction of growth phase-dependent translational regulation in haloarchaea *in vivo*.

## Discussion

The biological roles of 5′-UTRs and 3′-UTRs of two selected *H. volcanii* genes have been studied *in vivo*. A reporter gene system was used that had been established recently to characterize translation initiation [22]. It turned out that it is also well suited analyze growth phase-dependent translational regulation. For both genes, which exemplify two opposite directions of differential translational control, it could be successfully demonstrated that the simultaneous presence of both 5′- and 3′-UTR is necessary and sufficient to transfer growth phase dependent translational regulation from the native transcript to the reporter transcript. In contrast, neither 5′- nor 3′-UTR alone are sufficient for regulation. This observation implies a functional interaction of the 5′- and 3′-UTR *in vivo*. To our knowledge this is the first such evidence for any bacterial or archaeal species.

In eukaryotes circularization of specific transcripts has been shown about 10 years ago [23], [24]. The circularization is thought to be caused by the common interaction of the cap-binding protein eIF4E and the polyA tail binding protein (PABP) with the scaffolding protein eIF4G. The ternary eIF4E/eIF4G/PABP complex has been reconstituted and using atomic force microscopy it could be shown that it is able to circularize capped, polyadenylated RNA [24]. Recently it was shown that the mammalian PABP is involved in key steps of translation initiation and might well be regarded as a canonical translation initiation factor [25]. Proteins interfering with the formation of the eIF4E/eIF4G/PABP complex induce translational repression and are involved in differential translational regulation [e.g. 26].

While our results indicate that at least functional interactions of 5′- and 3′-UTRs can also occur in haloarchaea, the mechanism must be different from the eukaryotic mechanism because haloarchaeal transcripts are neither capped nor polyadenylated. In addition, haloarchaea do not contain orthologs of eIF4E and PABP. Therefore, it seems that functional interactions of transcript ends has evolved prior to the development of mRNA capping and polyadenylation. Archaea contain a variety of translation initiation factors that are homologous to eukaryotic factors and that are not present in bacteria, which have only three initiation factors [review: 27]. It remains to be discovered if one or several of these archaeal initiation factors are involved in the functional interaction of 5′- and 3′-UTRs and translational regulation.

The UTR swap experiment has revealed that the direction of growth phase-dependent translational control is encoded in the 3′-UTR. Again, to our knowledge this is the first evidence for such a role of 3′-UTRs in any bacterial or archaeal species. In recent years it became apparent that translational control plays an important role also in bacteria. Regulation occurs at the translation initiation step, which is rate-limiting. The mechanisms typically involve sequences around the initiation site in the 5′-UTR, often differentially occluding the SD sequence from the ribosome [28]. Examples are RNA thermometers, riboswitches, sRNAs that need the Hfq protein to interact with the target RNA, and regulatory proteins that bind specific stemloop structures [reviews: 29]–[31].

In contrast to bacteria, regulation of translation by 3′-UTR binding factors is well studied in eukaryotes and numerous examples exist [13], [14], [32], [33]. A variety of regulatory RNA binding proteins have been identified, some of which belong to protein families that are widespread in eukaryotes. A bioinformatic search in the genome sequence of *H. volcanii* failed to identify homologs of conserved eukaryotic RNA binding proteins (present in *S. cerevisiae*, *S. pombe*, *A. thaliana*, *C. elegans*, human cell lines; data not shown). 3′-UTR binding proteins have not yet been identified in any other archaeal species. Therefore biochemical or genetic approaches will be required to identify haloarchaeal 3′-UTR-binding translational regulators and to elucidate the molecular mechanism of translational regulation.

## Materials and Methods

### Microorganisms, media, and growth conditions

*H. volcanii* WR 340 was obtained from Moshe Mevarech (Tel Aviv University, Tel Aviv, Israel) and *E. coli* XL1 Blue MRF' was purchased from Stratagene (Amsterdam, Netherlands). *H. volcanii* was grown aerobically in rich medium containing 2.9 M NaCl, 150 mM MgSO_4_, 60 mM KCl, 4 mM CaCl_2_, 0.275% (wt/vol) yeast extract, 0.45% (wt/vol) tryptone and 50 mM Tris-HCl, pH 7.2, at 42°C [34]. *E. coli* XL1 Blue MRF' was grown in SOB Medium at 37°C [35].

### Construction of plasmids containing 5′-UTR and 3′-UTR reporter gene fusions

All plasmids used in this study and their characteristic features are listed in Table 1. The shuttle vector pSD1/M2–18 [36] was used for the generation of a reporter system to study the *in vivo* function of 5′-UTRs and 3′-UTRs. It contains replication origins and resistance genes for *E. coli* and *H. volcanii* as well as the *dhfr* gene under the control of a constitutive promoter of medium strength, which allows detection of both up- and downregulation of expression levels.

The control plasmid pMB1 (No. 1 in Figure 2, 3, 5 and 5) and the reporter gene fusions pMB3 (No. 2), pMB4 (No. 3), pMB6 (No. 5) and pMB7 (No. 6) were constructed as described previously [22]. The plasmid pMB5 (No. 4) was constructed in a similar way. The promoter fragment and the ORF were amplified as separate PCR fragments and joined by fusion PCR. The 5′-UTR was part of the primers (a Table with the oligonucleotides used for the constructions is available upon request). The 3′-UTR and a *Kpn*I site were part of the downstream primer for amplification of the ORF fragment. For construction of plasmid pMB8 (No. 7) three PCR fragments were generated containing the promoter region with the 5′-UTR, the ORF, and the 3′-UTR, respectively. The three fragments were joined into one fragment by two consecutive fusion PCRs. The plasmid pMB23 (No. 11) were generated as described above for pMB5. The plasmids pMB22 (No. 10), pMB24 (No. 8) and pMB25 (No. 9) were constructed as described above for pMB8.

In all cases, the final fragments were cloned into the shuttle vector pSD1/M2–18 using single *Apa*I and *Kpn*I sites. The newly generated regions of all plasmids were verified by sequencing. Then they were used to transform *H. volcanii* as described previously [34].

### Determination of *dhfr* transcript levels

RNA was isolated from exponentially (2×10^8^ cells/ml) and stationary (2×10^9^ cells/ml) growing cultures as described by Chomczynski and Sacchi [37]. DNase treatment, reverse transcription and Real-time PCR analysis were performed as described previously [22].

As an unregulated control, the *hpyA* transcript levels were determined with the primer pair hpyA-RT_f and hpyA-RT_r. The ΔΔC_t_ method [38] was used for the analysis of the Real-time PCR results. The C_t_ levels of the control transcript *hpyA* were used to normalize the C_t_ levels of the *dhfr* transcripts. The *dhfr* level of the chromosomal gene copy was determined using a strain carrying a plasmid without a *dhfr* gene (pNP10) [39]. It was only a small fraction of the total *dhfr* transcript level, nevertheless the value was subtracted to quantitate the transcript level of the plasmid-encoded *dhfr* gene. The *dhfr* transcript level of the strain containing pMB1, which encodes a *dhfr* without 5′-UTR and 3′-UTR, was set to 1.

### Determination of DHFR activities and of translational efficiencies

The determination of the DHFR activities and translational efficiencies for *H. volcanii* cultures from exponential (2×10^8^ cells/ml) and stationary (2×10^9^ cells/ml) growth phase were performed as described previously [22].

Alternatively DHFR activity was measured in 250 µl volume containing 50 µl of cytoplasmatic extract, 167.5 µl buffer (3 M KCl, 25 mM potassium phosphate, 25 mM citrate pH 6.0), 0.05 mM dihydrofolic acid (Sigma, Taufkirchen, Germany), and 0.08 mM NADPH (AppliChem, Darmstadt, Germany). The oxidation of NADPH was determined at 340 nm and 25°C with a SPECTRAmax 340PC_384_ photometer (Molecular Devices, Sunnyvale, USA). The enzymatic activity was calculated according to Brenneis et al. [22].

The DHFR level encoded by the chromosomal *dhfr* copy was determined using a strain carrying a plasmid without a *dhfr* gene (pNP10) [39]. Again, it was only a small fraction of the total DHFR level of reporter gene-containing strains, but it was subtracted to quantitate the plasmid-encoded DHFR level. The translational efficiencies were calculated by dividing the specific enzyme activities with the transcript levels. At least three independent experiments were performed, and average values and standard deviations were calculated.

### Prediction of secondary structures

The program “Mfold 3.2” at the website http://www.bioinfo.rpi.edu/applications/ mfold/rna/form1.cgi was used for the prediction of the possible secondary structures of 5′-UTRs, 3′-UTRs and complete transcripts [40], [41].
